# Climate crisis risks to elderly health: strategies for effective promotion and response

**DOI:** 10.1093/heapro/daae031

**Published:** 2024-04-03

**Authors:** Mahsa Madani Hosseini, Manaf Zargoush, Somayeh Ghazalbash

**Affiliations:** Ted Rogers School of Management, Toronto Metropolitan University, Toronto, ON, *M5B 2K3*, Canada; Health Policy & Management, DeGroote School of Business, McMaster University, Hamilton, ON, L8S 4M4, Canada; Management Analytics, Smith School of Business, Queen’s University, Kingston, ON, K7L 3N6, Canada

**Keywords:** climate crisis, older adults, disasters, risk management, health promotion

## Abstract

The climate crisis significantly impacts the health and well-being of older adults, both directly and indirectly. This issue is of growing concern in Canada due to the country’s rapidly accelerating warming trend and expanding elderly population. This article serves a threefold purpose: (i) outlining the impacts of the climate crisis on older adults, (ii) providing a descriptive review of existing policies with a specific focus on the Canadian context, and (iii) promoting actionable recommendations. Our review reveals the application of current strategies, including early warning systems, enhanced infrastructure, sustainable urban planning, healthcare access, social support systems, and community engagement, in enhancing resilience and reducing health consequences among older adults. Within the Canadian context, we then emphasize the importance of establishing robust risk metrics and evaluation methods to prepare for and manage the impacts of the climate crisis efficiently. We underscore the value of vulnerability mapping, utilizing geographic information to identify regions where older adults are most at risk. This allows for targeted interventions and resource allocation. We recommend employing a root cause analysis approach to tailor risk response strategies, along with a focus on promoting awareness, readiness, physician training, and fostering collaboration and benchmarking. These suggestions aim to enhance disaster risk management for the well-being and resilience of older adults in the face of the climate crisis.

**Contribution to Health Promotion**

- This study contributes to knowledge on effective health promotion and resilience among older adults in the face of climate crisis.
- Current practices are inadequate for mitigating the impacts of climate crisis on older adults within the Canadian context.
- A series of strategic recommendations should be put forth to enhance the health and resilience of older adults facing the climate crisis.
- We emphasize the importance of robust risk metrics, vulnerability mapping, root cause analyses, awareness and preparedness, physician training, and collaboration.

## INTRODUCTION

Climate change constitutes a crisis (Zeldin-O’Neill, 2019) driven by complex interplays of commercial (Arnot *et al.*, 2023a; Gilmore *et al.*, 2023), political (Yeganeh *et al.*, 2020; Arnot*et al.*, 2023; Arnot *et al.*, 2023b) and social (Dankelman and Naidu, 2020; Klinenberg *et al.*, 2020; Gislason *et al.*, 2021) factors, demanding immediate attention and action. Commercial activities, particularly in the fossil fuel industry, significantly drive the climate crisis through mechanisms including supply chains, industrial processes, global trade, transportation and resource extraction (Gilmore *et al.*, 2023). These sectors release substantial carbon dioxide, intensifying the greenhouse effect and global warming. Profit motives also fuel excessive consumption and hinder sustainability (Arnot *et al.*, 2023b). Political determinants, like government regulations and international agreements, are vital for climate action but are often influenced by economic imperatives, necessitating urgent responses to the climate crisis. Social determinants emphasize societal factors (e.g. income, infrastructure) and behaviors (e.g. cultural practices) in the climate crisis (Ercan *et al.*, 2022).

The climate crisis poses a significant threat to human health, with the World Health Organization (WHO) estimating an additional 250 000 deaths annually between 2030 and 2050 (WHO, 2014). It impacts human well-being both directly and indirectly. Direct impacts include increased mortality and illnesses caused by extreme climate events such as heat waves, storms, wildfires, floods and droughts. Indirect effects result from changes in ecosystems, economies and social structures, leading to the spread of disease vectors, waterborne pathogens and deterioration in air, water and food quality (Watts *et al.*, 2015; Caminade *et al.*, 2019).

The impacts of climate crisis on elderly people are particularly pressing in Canada due to several reasons. First, the impacts of the climate crisis on human health are significantly influenced by age, with older adults being at a higher risk of adverse effects (Fulop *et al.*, 2010; Gamble *et al.*, 2013; Perez *et al.*, 2022). The situation becomes more complex due to the health inequalities related to the climate crisis concerning older adults, which primarily stem from disparities in vulnerability and resilience. For instance, a significant portion of older adults in Canada live alone (Statistics Canada, 2012), have disabilities (Arim, 2015), and suffer from chronic conditions (Carstairs, 2000), with many experiencing financial stress (Campbell and Keehn, 2018). Second, Canada is experiencing a faster rate of warming compared to the global average (Lewis and Dickson, 2019; Public Health Agency of Canada, 2019). Lastly, the older adult population in Canada is projected to increase from 15% in 2011 to 25% in 2036, posing a significant threat to the health of this vulnerable demographic (Statistics Canada, 2011; United Nations, 2017).

The above discussion underscores the importance of raising awareness and improving our understanding of how climate crisis affects older adults. This article has three main objectives: (i) to outline the direct and indirect effects of climate crisis on older adults, (ii) to provide a descriptive review of existing policies with a specific focus on the Canadian context and (iii) to propose recommendations to enhance current practices in mitigating the impacts of the climate crisis on older adults.

## IMPACTS OF CLIMATE CRISIS ON ELDERLY HEALTH

**Fig. 1:**
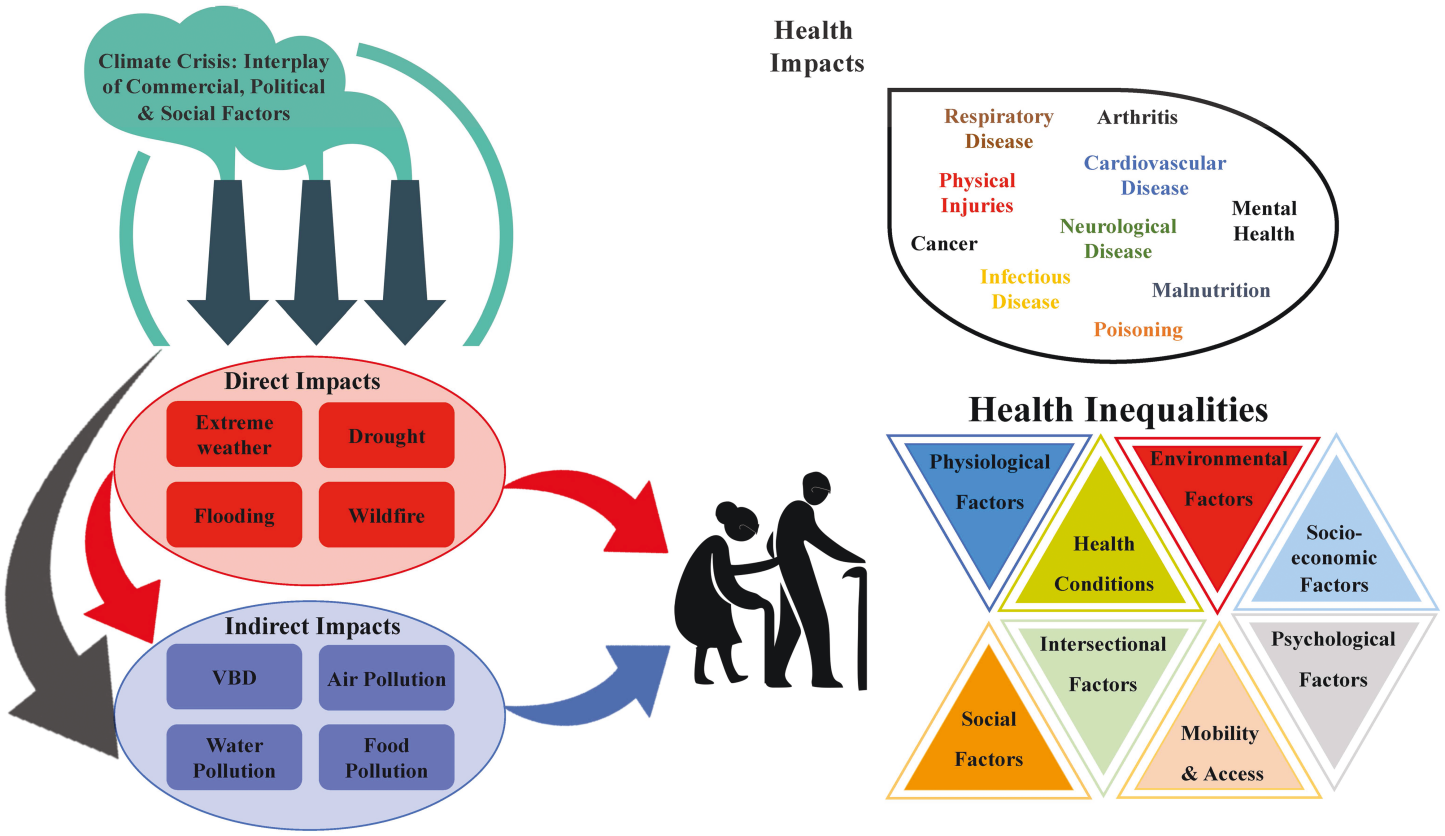
Health inequalities and impacts of climate crisis on elderly.

In this section, we explore the impacts of climate crisis on older adults’ health (Figure 1). The complication arises from health inequalities, placing older adults at a higher risk in the face of the climate crisis. This concern is increasingly recognized in health promotion and public health priorities (Thomas and Daube, 2023; Thomas *et al.*, 2024).

Health disparities among older adults due to climate change stem from vulnerability and resilience factors. Aging reduces thermoregulation and weakens immunity, heightening risks during climate events. Socioeconomic disparities limit access to essentials like air conditioning and healthcare, hindering adaptation. Existing health issues worsen outcomes during heat waves and air pollution. Geographical factors in disaster-prone areas intensify health risks, compounded by sparse vegetation. Social isolation and limited information hinder preparation, while disabilities impede access. Intersectional factors like race, ethnicity, gender and sexual orientation exacerbate disparities. Minorities face higher risks due to socioeconomic gaps, and LGBTQ+ elders may face discrimination during disasters (Goldsmith *et al.*, 2022).

### Direct impacts

The climate crisis directly impacts human health, especially through extreme weather events like heat waves, floods, droughts and wildfires. These events pose significant health risks for older adults. The following sections will examine each of these impacts individually, focusing on Canada.

#### Extreme weather

The Earth experiences more extreme weather due to climate change, with rising heat waves and the potential for colder temperatures (Kim *et al.*, 2017; Gibbens, 2024). In Canada, addressing heat wave-related risks is prioritized (Beugin *et al.*, 2023), supported by significant warming in recent decades, with Canada warming at twice the global rate (Government of Canada, 2023). Canadians have adapted to extreme cold through physiological changes (Alberini *et al.*, 2011; Haman *et al.*, 2022), leading to concerns about increased heat-related mortality, especially in cities like Hamilton. Projected increases of 7%, 15% and 25% for 2031–2050, 2051–2070 and 2071–2090, respectively, compared to 2011 mortality rates, highlighting the urgency of addressing heat-related risks (Martin *et al.*, 2012).

Numerous studies have linked rising temperatures to increased morbidity and mortality (Yang *et al.*, 2019; Oudin Åström *et al.*, 2020; Royé *et al.*, 2020; Zhang *et al.*, 2021), particularly among older adults (Li *et al.*, 2015; Zhang *et al.*, 2015; van Steen *et al.*, 2019; Diniz *et al.*, 2020; Wilk *et al.*, 2021). For instance, the risk of cardiovascular and respiratory mortality increases on heat wave days compared to nonheat wave ones (Silveira *et al.*, 2023). The health disparities exacerbating heat wave impacts include age-related vulnerability, social isolation (Kim *et al.*, 2020), low income and education (Green *et al.*, 2019), lack of cooling systems, sparse vegetation, urbanization (Burkart *et al.*, 2016; Chen *et al.*, 2016) and chronic diseases (Hopp *et al.*, 2018). Older adults in Canada are disproportionately affected by various factors. Over 50% experienced social isolation and financial challenges (Statistics Canada, 2017), and 40% lacked air conditioning in 2019 (Statistics Canada, 2021). While 60% live in urban areas, 40% reside in rural regions (Channer *et al.*, 2020). Additionally, 80% have at least one chronic disease, impacting their quality of life (Carstairs, 2000). Chronic diseases increase the risk of heat-related illness, posing challenges during extreme heat events (Semenza *et al.*, 1999; Ishigami *et al.*, 2008; Meade *et al.*, 2020). These conditions, prevalent among older adults, impair their physiological responses and diminish their ability to adapt to changes in environmental conditions.

#### Flooding

The heightened frequency and intensity of extreme weather events, such as flooding, pose significant risks to older adults. Direct consequences include death and injuries, while indirect impacts encompass mental health issues, waterborne illnesses and respiratory/cardiovascular diseases (Shrubsole, 2013; Sahni *et al.*, 2016; Pelletier, 2017). Flooding is expected to become a major climate-related threat in Canada, with 22 events from 2000 to 2015 resulting in 18 deaths and affecting 126 474 people (Burton *et al.*, 2016). Older adults face *health inequalities* related to residential location and healthcare access, particularly in communities near water bodies (Shrubsole, 2013; Council of Canadian Academies, 2019). Their vulnerability is compounded by social isolation, limited awareness of flood risks, disabilities and reduced physical capabilities (Adams *et al.*, 2011). These factors increase susceptibility to immediate dangers and exacerbate long-term health issues, including stress and mental health conditions (Sahni *et al.*, 2016; Sands *et al.*, 2022).

#### Droughts

Elevated temperatures from global warming exacerbate drought’s impact on vulnerable groups, notably older adults. Severe drought is observed in southern Canadian Prairies and British Columbia’s interior valleys. Research demonstrates that those aged 65 and older, particularly 75 and above, face heightened mortality risks from drought (Salvador *et al.*, 2021, 2022). This heightened vulnerability arises from a combination of *disparity factors*. Physiological changes associated with aging, such as weakened immune systems, make older adults more susceptible to waterborne and airborne diseases. Existing health conditions and medications can exacerbate these vulnerabilities, while social and economic factors like isolation and limited income can hinder their ability to adapt to drought conditions. Psychological impacts, such as stress and anxiety, further compound these challenges (Horton *et al.*, 2010). Drought also has numerous indirect impacts on human health, including waterborne illnesses (Yusa *et al.*, 2015; Hyllestad *et al.*, 2020), airborne dust-related diseases (Stanke *et al.*, 2013; Yusa *et al.*, 2015), undernutrition and malnutrition (Lieber *et al.*, 2022), mental health problems (Vins *et al.*, 2015) and an increased risk of infectious and vector-borne diseases (VBD) (Yusa *et al.*, 2015).

#### Wildfires

The climate crisis heightens the risk of wildfire ignition sources and the growth of flammable vegetation, resulting in around 8000 annual wildfires in Canada (EPA, 2021). In 2023, Canada experienced record-breaking wildfires, scorching an area larger than Greece (Milman, 2023). In Canada, older adults face heightened vulnerability to wildfires due to various health disparities. These include insufficient awareness of wildfire risks, often linked to limited access to timely and comprehensible information. Challenges during emergency evacuations are amplified by physical constraints, absence of personal transportation and social isolation. Wildfire smoke exposure significantly exacerbates health conditions, resulting in increased respiratory and cardiovascular ailments among older adults (Ye *et al.*, 2021). Moreover, the stress and trauma induced by wildfires can trigger mental health issues, particularly anxiety and depression, among this demographic (DeFlorio-Barker *et al.*, 2019; To *et al.*, 2021).

### Indirect impacts

The climate crisis has emerged as a significant global concern, with far-reaching indirect impacts on the health of older individuals. As we discuss below, these impacts have been observed and supported by compelling evidence, particularly in the context of Canada.

#### Vector-borne diseases

The climate crisis, resulting in elevated temperatures, has amplified Lyme disease (LD) occurrences in Ontario, Canada (Cheng *et al.*, 2017), which may manifest as flu-like, arthritic, neurological and cardiac symptoms (Johnson *et al.*, 2018). Older adults with less effective immune systems confront heightened LD susceptibility and suboptimal treatment outcomes (Boršič *et al.*, 2018; Zomer *et al.*, 2018). Furthermore, reported human infections of West Nile virus (WNV) notably surged from 2014 to 2018, with the highest incidence recorded in the Canadian prairie provinces (Chen *et al.*, 2013; Public Health Agency of Canada, 2019). Severe WNV infections can lead to long-term physical issues, mental health issues, neurological diseases and death (Sejvar, 2014; Bai *et al.*, 2019). In 2018, most of the 29 WNV-related deaths occurred among infected older Canadian adults (Public Health Agency of Canada, 2018). Socioeconomic and geographical disparities can worsen the effects of VBDs in older adults. Limited financial resources and healthcare access increase their vulnerability to severe health consequences. Rural or underfunded regions, which are at greater risk for disease exposure, often lack adequate medical resources for prompt diagnosis and treatment. These inequalities create a troubling cycle where older adults, who are already vulnerable due to weakened immune systems, face elevated risks and suboptimal treatment outcomes.

#### Water, air and food pollution

The climate crisis, with heavy rainfall and rising temperatures, negatively impacts water and air quality, straining Canada’s water treatment systems (Chhetri *et al.*, 2019). This increases the risk of waterborne illnesses among older adults. Proactive adaptation and strengthening of water systems are crucial to mitigate climate crisis-related health risks in this vulnerable group.

Disparities in infrastructure (e.g. outdated water treatment systems), healthcare access and socioeconomic status increase older adults’ vulnerability to climate-related waterborne, air and foodborne diseases. These disparities, coupled with preexisting health conditions and weakened immunity, create a cycle of heightened vulnerability and poorer health. For instance, older adults, due to preexisting health conditions, face heightened vulnerability to air pollution (Simoni *et al.*, 2015), resulting in various health issues, including respiratory problems (Lepeule *et al.*, 2014), peptic ulcer bleeding (Tian *et al.*, 2017), Alzheimer’s disease (Wu *et al.*, 2015), chronic kidney disease (Chen *et al.*, 2018) and cardiovascular disease (Chuang *et al.*, 2011). With the climate crisis, the mortality rate linked to air pollution is expected to soar, especially among older adults (Canadian Medical Association, 2008).

Climate crisis increases the risk of foodborne diseases through flooding and rising temperatures, impacting diseases like *Campylobacter*, *Escherichia coli* and *Salmonella*. Floods can contaminate fields with pesticides and animal waste, while higher temperatures expand disease-carrying wildlife vectors (Smith *et al.*, 2019). In Canada, these diseases already affect millions, resulting in hospitalizations and deaths (Thomas *et al.*, 2013). Older adults are especially vulnerable due to weakened immune systems, chronic illnesses, malnutrition, dementia and unsafe food practices (Lake, 2017; Smith *et al.*, 2019).

## REVIEW OF MITIGATION STRATEGIES AND ACTION PLANS

Efforts to minimize disaster impact on vulnerable populations are based on four pillars: prevention/mitigation, preparedness, response and recovery (Field *et al.*, 2012). Table 1 outlines existing disaster risk management policies for the elderly, addressing the direct and indirect impacts of the climate crisis. In the following sections, we will examine these policies and corresponding strategies for older adults.

**Table 1:** Risk management actions associated with direct and indirect impacts of climate crisis

	Risk management strategies			
Disaster	Prevention/mitigation	Preparedness	Response	Recovery
Heat wave	– Preparing emergency response plan – Vulnerability mapping – Land transformation – Housing stock – Urban planning – Energy use	– Public awareness and education	– Opening of 24-h cooling center – Transferring older adults to cooling centers – Extreme weather alert	– Medical care and monitoring – Social support and companionship
Flooding	– Preparing emergency response plan – Flood maps – Land use planning – Predicting and warning of floods	– Public awareness and education – Backup supply of medicine, food and water	– Sandbags distribution – Evacuation planning – Accessibility and mobility – Coordination and collaboration	– Psychological assistance – Financial assistance
Drought	– Preparing response plan – Water resource management – Crop management – Vulnerability assessment	– Education and awareness – Advance prediction – Early warning system	– Increase water supplies – Reduce water use – Health monitoring – Medical support – Coordination and collaboration	– Psychological assistance – Financial assistance
Wildfire	– Fire-resistant buildings – Vegetation management	– Advance prediction – Education & awareness – Support networks	– Early detection systems – Evacuation assistance – Care facilities access	–Psychological assistance – Financial assistance – Community engagement
Vector-borne diseases (LD and WNV)	– Preparing prevention plan – Surveillance and public awareness – Government regulations – Control of mosquito population	– Public awareness and education	– Diagnostic test – Treatment – Supportive services	– Rehabilitation services – Pain management – Emotional support
Air, water, and food pollution and related disease	– Air/water/food surveillance – Improving water treatment plants – Air pollutant emission control – Enhanced food inspection and regulation	– Public awareness and education – Developing early warning systems and alerts – Community-based support networks	– Establishing communication channels – Food recall – Access to clean water – Diagnostic test – Treatment	– Health monitoring and treatment – Rehabilitation services – Psychological assistance

### Policy approaches to mitigate the impact of extreme weather

In Canada, the government has historically implemented measures to address cold-related challenges, including offering energy home grants to improve heating systems and reduce energy waste during winter. For example, the Government of Ontario’s Energy Rebates program provides substantial incentives for energy-efficient practices, such as offering up to $325 for each high-performance window upgrade, with total rebates reaching $10 000. These initiatives aim to help Canadians deal with the challenges of cold waves during winter. However, it is crucial to acknowledge that while efforts to tackle cold-related issues are in place and actively promoted, the increasing threat of heat-related deaths demands a renewed and robust response.

Effective heat-related condition prevention requires comprehensive emergency response plans. Identifying responsible agencies like public health and fire authorities, the Canadian Red Cross and Meals on Wheels is vital (Lubik and Kosatsky, 2017). Vulnerability mapping, considering factors like air conditioning availability and age demographics, identifies high-risk areas (Reid *et al.*, 2009; Schmeltz *et al.*, 2013). While air conditioning is crucial for elderly health during heat waves, acknowledging its sustainability challenges is essential (Prek, 2004; Grignon-Masse *et al.*, 2011). Promoting energy-efficient cooling technologies and responsible air conditioning practices can address elderly needs while minimizing environmental impact. Implementing long-term measures like urban vegetation, heat-resilient infrastructure and improved cooling access is crucial (Burkart *et al.*, 2016; Taylor *et al.*, 2018, 2021). Collaborative efforts of public health experts, urban planners and stakeholders are vital to mitigating urban heat islands in Canadian cities (Health Canada, 2020).

To enhance heat wave *preparedness* and *response*, effective communication and public education are vital (Singh *et al.*, 2015). This includes promoting individual actions like increased fluid intake and reduced physical activity (Dotto *et al.*, 2010). Community measures such as opening cooling centers, extending pool hours and providing heat warnings are essential. Activation and deactivation criteria based on humidity and temperature triggers are crucial (Lubik and Kosatsky, 2017). While Canada has heat alerts and response systems, their effectiveness for older adults with impairments needs evaluation. Poorly coordinated responses to extreme weather harm vulnerable populations (Benevolenza and DeRigne, 2019).

Effective *recovery* measures are therefore crucial, including medical care, monitoring and social support (Benevolenza and DeRigne, 2019). Healthcare professionals can provide necessary treatments, while community outreach and volunteers provide emotional support, combat isolation and connect older adults with essential resources during heat waves.

### Policy approaches to mitigate the impact of flooding

To *mitigate* flood impacts, emergency plans must consider health inequalities affecting older adults, including their health status, access to healthcare, transportation and the potential for increased hospital visits (Burton *et al.*, 2016). Unfortunately, over 25% of Ontario hospitals lacked disaster risk management mechanisms (Seeman *et al.*, 2008). Therefore, a robust mitigation strategy, which may include land use planning, flood mapping and warning systems, collectively helps reduce flood impacts (Government of Ontario, 2020). For example, effective land use planning may recommend increased vegetation to enhance water absorption capacity, thereby reducing runoff and the risk of flooding.

The 2013 Alberta floods revealed insufficient flood *preparedness*, emphasizing the need to enhance readiness (Austin *et al.*, 2015). This involves training older adults and caregivers, bolstering caregiver capacity and stockpiling vital supplies. Such measures are vital as floods can disrupt crucial services and access to necessities (Yodsuban and Nuntaboot, 2021).

Evacuating older adults during floods often neglects their specific needs, emphasizing the importance of equitable *response* measures (Bukvic *et al.*, 2018). This entails prioritizing accessibility and appropriate infrastructure for those with mobility challenges (Ashida *et al.*, 2016; Brockie and Miller, 2017). Incidents like seniors encountering deep waters in Ottawa (Westwood, 2017) and discomfort in crowded facilities in Calgary (Muscedere and Heckman, 2019) highlight the necessity for coordinated resources, information sharing and partnerships among government agencies, healthcare providers and community groups.

Financial concerns affect approximately 50% of Canadians aged 60 and over (Campbell and Keehn, 2018). Therefore, in disaster *recovery* planning, prioritizing psychological and designing *targeted financial support* is crucial (Thompson *et al.*, 1993). To address this, governments should provide financial aid to seniors affected by disasters like floods. For instance, the Ontario government has recently pledged support to flood victims, emphasizing the importance of such aid for seniors’ recovery (Government of Ontario, 2020).

### Policy approaches to mitigate the impact of drought

The primary *preventive* and *mitigation* strategies to combat drought involve vulnerability assessment and water resource management, including rainwater harvesting and reservoir construction (Gautam and Bana, 2014). Collaboration among stakeholders, including farmers and water management agencies, is crucial to develop integrated drought management plans. Promoting drought awareness among stakeholders, including farmers, is vital, alongside physical *preparedness* measures and educational programs about sustainable water use, drought-resistant farming and conservation (Gautam and Bana, 2014). In addition, early warning systems and risk assessments empower communities for preparedness (Nhamo *et al.*, 2019). *Responses* include promoting water conservation and enforcing use restrictions through education campaigns (Yusa *et al.*, 2015). Initiatives like the Invitational Drought Tournament in British Columbia aim to manage drought impacts through monitoring, education and mental health programs (Hill *et al.*, 2014). Health monitoring systems for older adults during drought are crucial, including access to medical care and guidance on managing heat stress. *Recovery* plans should involve establishing drought response teams, securing funding and fostering coordination among government agencies, healthcare providers and community groups (EPA, 2016).

### Policy approaches to mitigate the impact of wildfire

To *mitigate* wildfires, regulations should minimize human-induced fires, utilizing resource allocation, fire permit restrictions and equipment rules. Decision support tools are, therefore, essential for efficient wildfire management (Tymstra *et al.*, 2020). Educating older adults about creating defensible spaces by removing flammable materials and maintaining vegetation is also critical in wildfire mitigation.

Educational initiatives play a pivotal role in *preparedness* plans by raising awareness among older adults about wildfire risks and health consequences. For instance, recent guidelines in the United States advise seniors to maintain essential supplies, including medications, N95 masks and portable air cleaner filters (*HomeInstead*, 2023). Social cohesion has been shown to enhance resilience to wildfires, ultimately reducing their health effects (Prior and Eriksen, 2013).

An effective wildfire *response* strategy necessitates comprehensive education, including outreach programs and educational materials for older adults about wildfire risks, evacuation protocols and early detection. Encouraging them to create evacuation plans, identify routes, prepare emergency kits and establish communication networks with family, neighbors and authorities is crucial.

The *recovery* strategies are essential to restoring the emotional, financial and physical well-being of older adults affected by climate change disasters (Rosenthal *et al.*, 2021). Strategies include postdisaster counseling, support groups and mental health services for emotional and psychological impacts. Financial assistance aids in home repairs, insurance claims and accessing benefits. Community engagement and volunteer efforts assist older adults in rebuilding and resource access (Corbin *et al.*, 2021).

### Policy approaches to mitigate the impact of VBDs

Climate change exacerbates existing inequalities, including those resulting from economic injustice (Islam and Winkel, 2017). Poverty, inadequate housing and poor environmental conditions increase vulnerability to VBDs, especially among children, women and older adults (Sutherst, 2004; Thomson and Stanberry, 2022). Targeted financial support for surveillance and control is, therefore, an essential *preventive* measure to enhance public awareness, detection, prevention and treatment of VBDs (Organization, 2019). Financial support for community-based strategies is also crucial, involving public policies regulating land use and construction, as well as surveillance for vectors and disease incidence. Early warning systems can improve surveillance effectiveness, facilitating prompt response measures such as public awareness campaigns (Thomson and Stanberry, 2022). Other *preventive* measures include monitoring tick distribution, effective tick habitat management and personal protective measures (Harymann *et al.*, 2014; de la Fuente, 2018; Eisen, 2018; Moon *et al.*, 2019; van Steen *et al.*, 2019; Bron *et al.*, 2020).

Increasing public awareness of tick-borne diseases is vital for enhancing *preparedness* to facilitate timely testing and pathogen identification (Scott and Scott, 2018; Hornbostel *et al.*, 2021). *Response* actions for individuals diagnosed with LD involve diagnostic tests and treatment, as there is currently no specific vaccine available for WNV (Rantakokko *et al.*, 2013; Harymann *et al.*, 2014; Johnson *et al.*, 2018). The *recovery* options for older adults facing VBDs include health monitoring, rehabilitation services, such as speech therapy, occupational therapy, or cognitive rehabilitation, pain management techniques and emotional support (Patel *et al.*, 2021; Scarciglia *et al.*, 2023).

### Policy approaches to mitigate the impact of air/water/food pollution and related diseases

*Preventive* measures for climate crisis-related pollution involve policy implementation, public awareness and quality monitoring (Eskander and Fankhauser, 2020). Canada utilizes various policies to address air pollution (Government of Canada, 2020; Newell, 2021), including carbon pricing, subsidies for cleaner technologies and emission standards enforcement. Vegetation plays a crucial role in pollutant removal and reducing respiratory issues. Moreover, regular food inspection systems and enforcing regulations are also crucial to ensure compliance with hygiene and safety standards (Barnes *et al.*, 2022).

*Preparedness* measures encompass environmental education and health risk awareness. Healthcare professionals play a crucial role in foodborne illness awareness education on air pollution. Early warning systems should notify older adults of elevated pollution levels, especially during heat waves when ground-level ozone production intensifies. These alerts should include guidance on protective measures. Additionally, community support networks are vital for disseminating information and resources, bolstering resilience and protecting the health of older adults (Kendall *et al.*, 2006; Radisic and Newbold, 2015; Karataş and Karataş, 2016).

Effective *responses* rely on timely air and water quality communication. The Public Health Agency of Canada uses environmental data to prevent gastrointestinal illnesses (Moffat and Struck, 2011). Investments in water systems and operator training have reduced waterborne diseases. Strengthening surveillance systems and research on mitigation measures are recommended (Smith and Fazil, 2019).

The effective *recovery* from pollution-related diseases relies on established health monitoring systems for timely risk identification and intervention, emphasizing regular checkups and assessments. Moreover, an inclusive recovery policy should cover physical well-being, offering resources and assistance such as rehabilitation programs for lingering effects or disabilities from pollution-related diseases. Mental health support is equally crucial due to its documented negative impact on mental well-being (Ramadan and Ataallah, 2021).

## A FRAMEWORK FOR ACTION: PROMOTING ELDERLY HEALTH PRACTICES

**Fig. 2:**
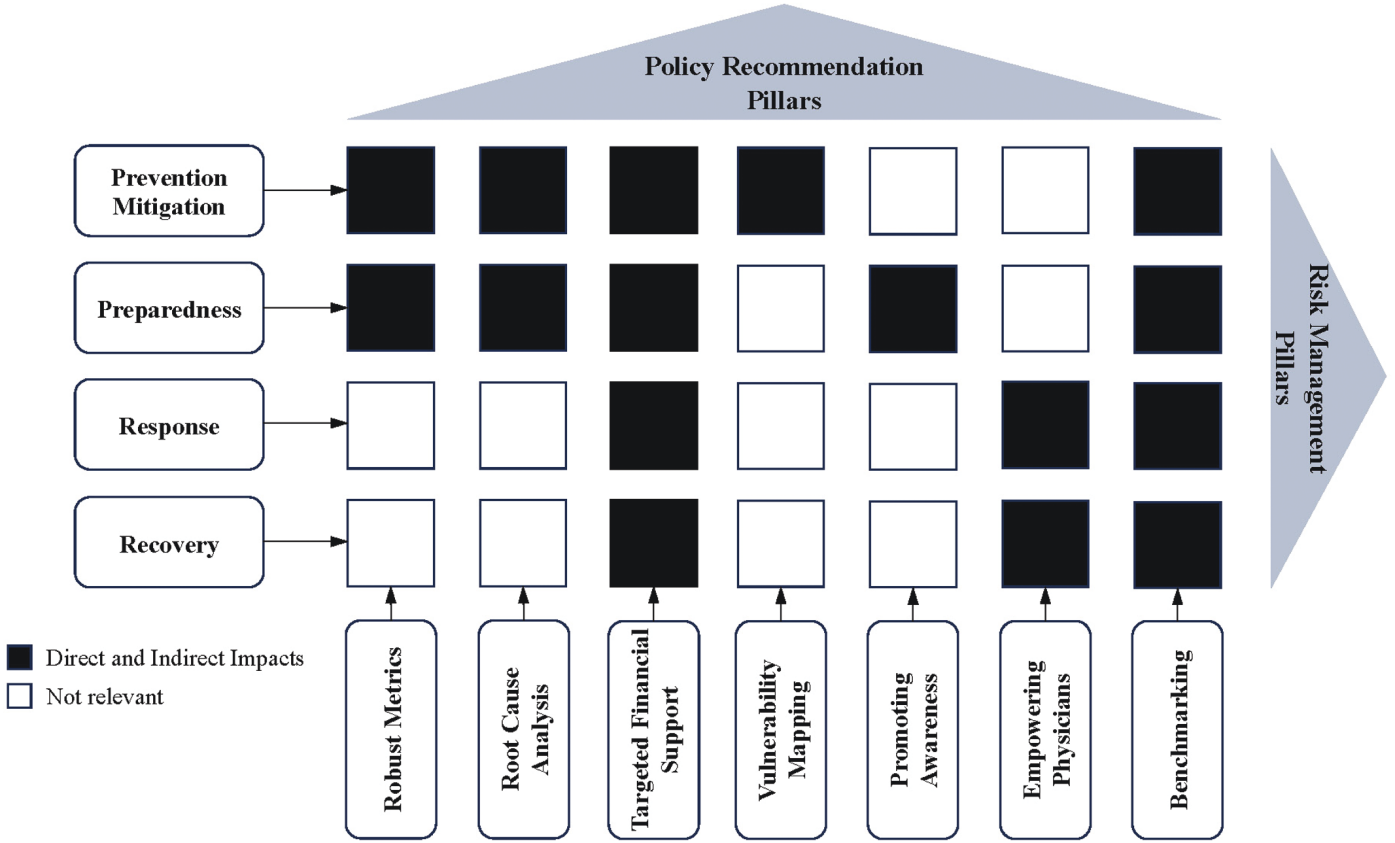
Policy recommendations for climate crisis impacts across four key risk management pillars.

Disaster risk management in Canada takes place at federal, provincial, territorial and municipal levels. However, there is a lack of focus on addressing the specific needs of older adults. Our review has identified strategies to improve the health of older adults in the face of climate crisis risks (Figure 2). It is essential to carefully consider the challenges faced by older adults when adapting to these risks.

### Robust risk metrics and assessment

The diverse impacts of the climate crisis on the elderly, coupled with budget constraints for risk response, call for *robust risk assessments* by health policymakers. This should include sensitivity analysis, stress testing, scenario analysis and consideration of extreme events. However, current Canadian research predominantly centers on heat waves, overlooking other climate-related disasters like floods and wildfires. The inadequate response to the 2013 Alberta floods and the air pollution from the 2023 Nova Scotia wildfires underscores the necessity for holistic risk management. To enhance resilience, Canadian organizations and policymakers should implement advanced early warning systems and emergency preparedness protocols, incorporating *cutting-edge technologies*, *real-time monitoring* (e.g. smartphone apps) and *community notification systems*.

### Root cause analysis to design tailored policies

Root cause analysis (RCA) plays a crucial role in developing tailored strategies for addressing climate change disasters, from reducing heat wave fatalities to managing wildfires in Canada. Despite advancements in heat wave prediction, the elderly in Canada remain highly vulnerable, with a severe heat wave in June 2021 causing 570 deaths in British Columbia, with 79% aged 65 or older (CBC News, 2021). RCA can identify key *predisaster* mitigation approaches by addressing the root causes of these impacts. For instance, if mortality is linked to a lack of cooling systems, the root cause may be seniors’ fixed incomes and rising costs. Government subsidies like funding or tax credits can promote energy-efficient cooling technologies and advocate for responsible air conditioning practices. Additionally, increasing green spaces in low-income neighborhoods can reduce heat risks, as wealthier areas typically have more green spaces. Implementing these measures improves access to cooling solutions and enhances urban environments, ultimately reducing heat-related fatalities.

RCA is crucial for addressing Canada’s increasing wildfires, exceeding the decade average by 20% (Cecco, 2023). RCA may reveal that wildfire origins extend beyond high temperatures, often involving human actions like deforestation and arson. Tailored mitigation can then be implemented, reallocating resources from firefighting to prevention efforts like controlled burns and public education.

### Targeted financial support

While increased financial support to mitigate climate impacts is crucial, it must effectively address the unique needs of the elderly, considering their health disparities. The ‘Canadian Climate Incentive’ exemplifies such initiatives; however, they often overlook climate justice and health inequalities, necessitating the inclusion of equity and human rights at the core of decision-making and action on climate change. Recent practices show that targeted financial support can improve climate justice, benefiting vulnerable populations, including children, older adults and people with disabilities or preexisting health conditions (EPA, 2023a). Another example involves addressing the concerns of local low-income BIPOC (Black, Indigenous and People of Color) communities, who face increasing risks from climate-exacerbated urban heat islands and coastal and inland flooding (EPA, 2023b). The research, funded by the US Environmental Protection Agency (EPA), aims to prioritize these concerns and propose climate resilience solutions, emphasizing the associated health benefits. Thus, while broad financial support is essential, focused initiatives tailored to older adults’ specific needs should be considered to improve equity and human rights, thereby having a more significant impact on mitigating climate risks for the vulnerable population.

### Vulnerability mapping with geographic information

Vulnerability mapping provides critical spatial data that decision support tools use to enhance risk management for climate change disasters in Canada by pinpointing high-risk areas for older adults and guiding targeted interventions. It is particularly valuable in coastal regions, like the Atlantic coast and the Beaufort Sea, for addressing flooding risks and planning evacuations. In heat wave-prone areas, like the south, it identifies neighborhoods lacking cooling resources, enabling cooling centers and fan distribution. Despite progress in Toronto and southern Quebec, provinces like Nova Scotia, British Columbia and Alberta should adopt such methods, exemplified by the 2023 Nova Scotia wildfires (Nova Scotia, 2023). Vulnerability mapping also addresses indirect impacts like VBDs, considering climate, population and environmental factors. This aids in identifying vulnerable areas for interventions such as mosquito control and awareness campaigns. Overall, vulnerability mapping is vital in reducing disease transmission risks associated with the climate crisis.

### Promoting awareness

Raising climate crisis awareness among older adults through media and the internet has limited effectiveness for those with dementia or limited computer skills. In Canada, there’s a shortage of guidelines for disaster preparedness in older adults. Research shows that many older adults lack emergency plans and preparedness information (Al-Rousan *et al.*, 2014). Despite available resources, a survey of 1304 older adults found that two-thirds lacked an emergency plan, had no disaster preparedness education, and were unaware of relevant resources. Over a third lacked essential supplies like food and water. These findings underscore the need to address these challenges and enhance awareness and preparedness among older adults, considering their limitations and ensuring accessible information and resources.

### Empowering physicians: a pathway to climate-resilient healthcare system

Training Canadian physicians to address climate-related health challenges is crucial for improving public health and eco-health literacy (Hackett *et al.*, 2020). The Canadian Association of Physicians for the Environment [CAPE (https://cape.ca)] plays a vital role in this regard, understanding the environmental impact on human health. Through educational initiatives, including an eight-module toolkit, CAPE empowers healthcare professionals and students to advocate for climate change policies (Patrick *et al.*, 2012; Perrotta, 2019). These efforts enable physicians to drive policy changes, engage communities, and enhance public awareness, contributing to a robust response to the climate crisis and reinforcing healthcare system resilience. For instance, CAPE advises heat response activities to protect health, including educating the public on protective measures, monitoring vulnerable populations and subsidizing improvements in the energy efficiency of housing for the elderly to reduce the pathological effects of energy poverty associated with increased incidence of respiratory problems and mental stress. Physicians can also aid research on climate crisis health impacts and response strategy effectiveness, informing evidence-based practices (Catford, 2008). They can improve patient outcomes, develop climate-sensitive treatment protocols and promote sustainable healthcare. Integrating climate crisis considerations enhances healthcare system resilience.

### Benchmarking: insights from collaborations

Collaborative efforts and benchmarking are essential for evidence-based climate crisis disaster management. They offer valuable insights, encourage knowledge sharing and support continuous improvement. Through benchmarking, we can evaluate *response* efforts, comparing factors like response times, coordination mechanisms, resource allocation and communication strategies across regions or organizations to identify areas for improvement. Similarly, benchmarking assesses *preparedness* by comparing emergency response plans, resource allocation, training programs and infrastructure, allowing for weaknesses to be identified and *mitigation* measures enhanced. An example of successful collaboration is the UK’s multi-institutional research program involving 14 universities, which developed a decision-based tool for extreme weather events (O’Neill *et al.*, 2009). Similar approaches can be applied in Canada, creating region-specific decision-based tools that integrate scientific knowledge and data-driven approaches to manage climate crisis disasters effectively.

## CONCLUSION

The climate crisis significantly impacts older adults in Canada, necessitating immediate action. This study reviews current strategies to mitigate direct and indirect effects on elderly health. These include enhancing early warning systems, healthcare infrastructure, urban planning, social support and community engagement. Tailored approaches involve relocating older adults during heat waves, ensuring flood resilience, managing drought-related water supply issues and proactive wildfire evacuation plans. The importance of medical emergency protocols and communication plans to address indirect impacts like VBDs is identified.

To enhance risk response plans, we propose several key actions. First, prioritize the establishment of *robust risk metrics*, including sensitivity, stress, scenario and extreme event analyses, to anticipate and manage unexpected climate change disasters effectively. Second, utilize *root cause analysis* to deepen understanding of underlying causes, followed by *targeted financial support* to tailor risk response strategies. Third, employ *vulnerability mapping*, using geographic data, to pinpoint areas where older adults are most vulnerable, facilitating targeted interventions and resource allocation. *Promote awareness* and preparedness among older adults, considering their unique limitations. *Empower physicians* with climate crisis training to advocate for policy changes and community engagement. Lastly, benchmarking with successful initiatives to inform evidence-based climate change disaster management approaches.

## References

[CIT0001] Adams, V., Kaufman, S. R., Van Hattum, T. and Moody, S. (2011) Aging disaster: mortality, vulnerability, and long-term recovery among Katrina survivors. Medical Anthropology, 30, 247–270. 21590581 10.1080/01459740.2011.560777 PMC3098037

[CIT0002] Alberini, A., Gans, W. and Alhassan, M. (2011) Individual and public-program adaptation: coping with heat waves in five cities in Canada. International Journal of Environmental Research and Public Health, 8, 4679–4701. 22408596 10.3390/ijerph8124679 PMC3290981

[CIT0003] Al-Rousan, T. M., Rubenstein, L. M. and Wallace, R. B. (2014) Preparedness for natural disasters among older US adults: a nationwide survey. American Journal of Public Health, 104, 506–511. 24432877 10.2105/AJPH.2013.301559 PMC3953784

[CIT0004] Arim, R. (2015) A *Profile of Persons with Disabilities among Canadians Aged 15 Years or Older, 2012*. Statistics Canada, Ottawa, Ontario. https://www150.statcan.gc.ca/n1/en/pub/89-654-x/89-654-x2015001-eng.pdf?st=7YJ_4kda (last accessed 29 Mrach 2024).

[CIT0005] Arnot, G., Pitt, H., McCarthy, S., Collin, P. and Thomas, S. (2023) Supporting young people as genuine political actors in climate decision-making. Health Promotion International, 38, 1–4. 10.1093/heapro/daad148 37952200

[CIT0006] Arnot, G., Thomas, S., Pitt, H. and Warner, E. (2023a) Australian young people’s perceptions of the commercial determinants of the climate crisis. Health Promotion International, 38, 10.1093/heapro/daad058 PMC10273831 37326409

[CIT0007] Arnot, G., Thomas, S., Pitt, H. and Warner, E. (2023b) Australian young people’s perspectives about the political determinants of the climate crisis. Health Promotion Journal of Australia, 35, 196–206. 37039480 10.1002/hpja.734

[CIT0008] Ashida, S., Robinson, E. L., Gay, J. and Ramirez, M. (2016) Motivating rural older residents to prepare for disasters: moving beyond personal benefits. Ageing & Society, 36, 2117–2140. 30013285 10.1017/S0144686X15000914 PMC6045947

[CIT0009] Austin, S. E., Ford, J. D., Berrang-Ford, L., Araos, M., Parker, S. and Fleury, M. D. (2015) Public health adaptation to climate change in Canadian jurisdictions. International Journal of Environmental Research and Public Health, 12, 623–651. 25588156 10.3390/ijerph120100623 PMC4306883

[CIT0010] Bai, F., Thompson, E. A., Vig, P. J. and Leis, A. A. (2019) Current understanding of West Nile virus clinical manifestations, immune responses, neuroinvasion, and immunotherapeutic implications. Pathogens, 8, 193. 31623175 10.3390/pathogens8040193 PMC6963678

[CIT0011] Barnes, J., Whiley, H., Ross, K. and Smith, J. (2022) Defining food safety inspection. International Journal of Environmental Research and Public Health, 19, 789. 35055611 10.3390/ijerph19020789 PMC8775694

[CIT0012] Benevolenza, M. A. and DeRigne, L. (2019) The impact of climate change and natural disasters on vulnerable populations: a systematic review of literature. Journal of Human Behavior in the Social Environment, 29, 266–281.

[CIT0013] Beugin, D., Clark, D., Miller, S., Ness, R., Pelai, R. and Wale, J. (2023) The Case for Adapting to Extreme Heat. Canadian Climate Institute, British Columbia. https://climateinstitute.ca/wp-content/uploads/2023/06/The-case-for-adapting-to-extreme-heat-costs-of-the-BC-heat-wave.pdf. (last accessed 29 March 2024).

[CIT0014] Boršič, K., Blagus, R., Cerar, T., Strle, F. and Stupica, D. (2018) Clinical course, serologic response, and long-term outcome in elderly patients with early Lyme borreliosis. Journal of Clinical Medicine, 7, 506. 30513820 10.3390/jcm7120506 PMC6306842

[CIT0015] Brockie, L. and Miller, E. (2017) Understanding older adults’ resilience during the Brisbane floods: social capital, life experience, and optimism. Disaster Medicine and Public Health Preparedness, 11, 72–79. 28095933 10.1017/dmp.2016.161

[CIT0016] Bron, G. M., Fernandez, M. P., Larson, S. R., Maus, A., Gustafson, D., Tsao, J. I. et al. (2020) Context matters: contrasting behavioral and residential risk factors for Lyme disease between high-incidence states in the Northeastern and Midwestern United States. Ticks and Tick-borne Diseases, 11, 101515. 32993935 10.1016/j.ttbdis.2020.101515

[CIT0017] Bukvic, A., Gohlke, J., Borate, A. and Suggs, J. (2018) Aging in flood-prone coastal areas: Discerning the health and well-being risk for older residents. International Journal of Environmental Research and Public Health, 15, 2900. 30567352 10.3390/ijerph15122900 PMC6313428

[CIT0018] Burkart, K., Meier, F., Schneider, A., Breitner, S., Canário, P., Alcoforado, M. J. et al. (2016) Modification of heat-related mortality in an elderly urban population by vegetation (urban green) and proximity to water (urban blue): evidence from Lisbon, Portugal. Environmental Health Perspectives, 124, 927–934. 26566198 10.1289/ehp.1409529 PMC4937850

[CIT0019] Burton, H., Rabito, F., Danielson, L. and Takaro, T. K. (2016) Health effects of flooding in Canada: a 2015 review and description of gaps in research. Canadian Water Resources Journal/Revue canadienne Des Ressources Hydriques, 41, 238–249.

[CIT0020] Caminade, C., McIntyre, K. M. and Jones, A. E. (2019) Impact of recent and future climate change on vector-borne diseases. Annals of the New York Academy of Sciences, 1436, 157–173. 30120891 10.1111/nyas.13950 PMC6378404

[CIT0021] Campbell, L. and Keehn, K. (2018) How to Help Seniors Avoid Financial Misery. https://www.theglobeandmail.com/business/commentary/article-how-to-help-seniors-avoid-financial-misery.

[CIT0022] Canadian Medical Association. (2008) No Breathing Room: National Illness Costs of Air Pollution. https://www.simcoemuskokahealth.org/docs/default-source/jfy-communities/pdf_jfy_municipality_enewsletter_feb2010_cma_icap_sum_e.

[CIT0023] Carstairs, S. (2000) A Roadmap for the Futureof Palliative Care in Canada. https://www.virtualhospice.ca/Assets/Raising%20the%20Bar%20June%202010_Senator%20Sharon%20Carstairs_20100608160433.pdf.

[CIT0024] Catford, J. (2008) Food security, climate change and health promotion: opening up the streams not just helping out down stream. Health Promotion International, 23, 105–108. 18492744 10.1093/heapro/dan016

[CIT0025] CBC News (ed.) (2021) 70% of sudden deaths recorded during B.C. heat wave were due to extreme temperatures, coroner confirms Social Sharing. https://www.cbc.ca/news/canada/british-columbia/bc-heat-dome-sudden-deaths-570-1.6122316#:~:text=Lisa%20Lapointe%20confirmed%20in%20an,with%20CBC's%20The%20Early%20Edition.

[CIT0026] Cecco, L. (2023) Canada faces ‘long, tough summer’ of wildfires with even hotter temperatures. The Guardian. https://www.theguardian.com/world/2023/jul/07/canada-wildfires-summer-weather-temps#:~:text=Canadian%20officials%20have%20warned%20that,shattering%20a%2034%2Dyear%20record (last accessed 29 March 2024).

[CIT0027] Channer, N. S., Hartt, M. and Biglieri, S. (2020) Aging-in-place and the spatial distribution of older adult vulnerability in Canada. Applied Geography, 125, 102357.

[CIT0028] Chen, C. C., Jenkins, E., Epp, T., Waldner, C., Curry, P. S. and Soos, C. (2013) Climate change and West Nile virus in a highly endemic region of North America. International Journal of Environmental Research and Public Health, 10, 3052–3071. 23880729 10.3390/ijerph10073052 PMC3734476

[CIT0029] Chen, K., Zhou, L., Chen, X., Ma, Z., Liu, Y., Huang, L. et al. (2016) Urbanization level and vulnerability to heat-related mortality in Jiangsu Province, China. Environmental Health Perspectives, 124, 1863–1869. 27152420 10.1289/EHP204 PMC5132638

[CIT0030] Chen, Q., Yu, Z., Yu, Z. and Wang, Z. (2018) The location decision of the elderly health club under the demand of maximum coverage. Journal of Discrete Mathematical Sciences and Cryptography, 21, 813–823.

[CIT0031] Cheng, A., Chen, D., Woodstock, K., Ogden, N. H., Wu, X. and Wu, J. (2017) Analyzing the potential risk of climate change on Lyme disease in eastern Ontario, Canada using time series remotely sensed temperature data and tick population modelling. Remote Sensing, 9, 609.

[CIT0032] Chhetri, B. K., Galanis, E., Sobie, S., Brubacher, J., Balshaw, R., Otterstatter, M. et al. (2019) Projected local rain events due to climate change and the impacts on waterborne diseases in Vancouver, British Columbia, Canada. Environmental Health, 18, 1–9. 31888648 10.1186/s12940-019-0550-y PMC6937929

[CIT0033] Chuang, K. -J., Yan, Y. -H., Chiu, S. -Y. and Cheng, T. -J. (2011) Long-term air pollution exposure and risk factors for cardiovascular diseases among the elderly in Taiwan. Occupational and Environmental Medicine, 68, 64–68. 20833756 10.1136/oem.2009.052704

[CIT0034] Corbin, J. H., Oyene, U. E., Manoncourt, E., Onya, H., Kwamboka, M., Amuyunzu-Nyamongo, M. et al. (2021) A health promotion approach to emergency management: effective community engagement strategies from five cases. Health Promotion International, 36, i24–i38. 34897448 10.1093/heapro/daab152 PMC8667549

[CIT0035] Council of Canadian Academies. (2019) Canada’s Top Climate Change Risks, Ottawa (ON): The Expert Panel on Climate Change Risks and Adaptation Potential. https://cca-reports.ca/wp-content/uploads/2019/07/Report-Canada-top-climate-change-risks.pdf (last accessed 29 March 2024).

[CIT0036] Dankelman, I. and Naidu, K. (2020) Introduction: gender, development, and the climate crisis. Gender & Development, 28, 447–457.

[CIT0037] de la Fuente, J. (2018) Controlling ticks and tick-borne diseases … looking forward. Ticks and Tick-borne Diseases, 9, 1354–1357. 29656834 10.1016/j.ttbdis.2018.04.001

[CIT0038] DeFlorio-Barker, S., Crooks, J., Reyes, J. and Rappold, A. G. (2019) Cardiopulmonary effects of fine particulate matter exposure among older adults, during wildfire and non-wildfire periods, in the United States 2008–2010. Environmental Health Perspectives, 127, 37006. 30875246 10.1289/EHP3860 PMC6768318

[CIT0039] Diniz, F. R., Gonçalves, F. L. T. and Sheridan, S. (2020) Heat wave and elderly mortality: historical analysis and future projection for metropolitan region of São Paulo, Brazil. Atmosphere, 11, 933.

[CIT0040] Dotto, L., Duchesne, L., Etkin, D. and Jaffit, E. (2010) Canadians at Risk: Our Exposure to Natural Hazards, Canadian Assessment of Natural Hazards Project. https://www.preventionweb.net/files/13008_CanadiansatRisk20101.pdf (last accessed 29 March 2024).

[CIT0041] Eisen, L. (2018) Pathogen transmission in relation to duration of attachment by *Ixodes scapularis* ticks. Ticks and Tick-borne Diseases, 9, 535–542. 29398603 10.1016/j.ttbdis.2018.01.002 PMC5857464

[CIT0042] EPA. (2016) Drought Response and Recovery, a Basic Guide for Water Utilities. United States Environmental Protection Agency. https://www.epa.gov/sites/default/files/2017-10/documents/drought_guide_final_508compliant_october2017.pdf (last accessed 29 March 2024).

[CIT0043] EPA. (2021) Which Populations Experience Greater Risks of Adverse Health Effects Resulting from Wildfire Smoke Exposure? United States Environmental Protection Agency, United States. https://www.epa.gov/wildfire-smoke-course/which-populations-experience-greater-risks-adverse-health-effects-resulting (last accessed 29 March 2024).

[CIT0044] EPA. (2023a) *Cumulative Health Impacts at the Intersection of Climate Change, Environmental Justice, and Vulnerable Populations/Lifestages: Community-Based Research for Solutions Grants*. United States Environmental Protection Agency, United States. https://www.epa.gov/research-grants/cumulative-health-impacts-intersection-climate-change-environmental-justice-and-3 (last accessed 29 March 2024).

[CIT0045] EPA. (2023b) *Advancing Community Resilience to Cumulative Climate Impacts in the Mystic River Watershed*. United States Environmental Protection Agency, April. https://cfpub.epa.gov/ncer_abstracts/index.cfm/fuseaction/display.abstractDetail/abstract_id/11364/report/0

[CIT0046] Ercan, E., Yatarkalkmaz, M. M. and Bulut, O. F. (2022) The impact of income level on household greenhouse gas emissions: a case study for Turkey. İstatistik Araştırma Dergisi, 12, 39–55.

[CIT0047] Eskander, S. M. and Fankhauser, S. (2020) Reduction in greenhouse gas emissions from national climate legislation. Nature Climate Change, 10, 750–756.

[CIT0048] Field, C. B., Barros, V., Stocker, T. F., Qin, D., Dokken, D.J., Ebi, K. L. et al. (2012) Managing the risks of extreme events and disasters to advance climate change adaptation: special report of the intergovernmental panel on climate change. https://www.ipcc.ch/report/managing-the-risks-of-extreme-events-and-disasters-to-advance-climate-change-adaptation/10.1136/jech-2012-201045 22766781

[CIT0049] Fulop, T., Larbi, A., Witkowski, J. M., McElhaney, J., Loeb, M., Mitnitski, A. et al. (2010) Aging, frailty and age-related diseases. Biogerontology, 11, 547–563. 20559726 10.1007/s10522-010-9287-2

[CIT0050] Gamble, J. L., Hurley, B. J., Schultz, P. A., Jaglom, W. S., Krishnan, N. and Harris, M. (2013) Climate change and older Americans: state of the science. Environmental Health Perspectives, 121, 15–22. 23033457 10.1289/ehp.1205223 PMC3553435

[CIT0051] Gautam, R. C. and Bana, R. S. (2014) Drought in India: its impact and mitigation strategies—a review. Indian Journal of Agronomy, 59, 179–190.

[CIT0052] Gibbens, S. (2024) *Why Cold Weather Doesn’t Mean Climate Change Is Fake*. National Geographic, January. https://www.nationalgeographic.com/environment/article/climate-change-colder-winters-global-warming-polar-vortex?loggedin=true&rnd=1710459971378

[CIT0053] Gilmore, A. B., Fabbri, A., Baum, F., Bertscher, A., Bondy, K., Chang, H. -J. et al. (2023) Defining and conceptualising the commercial determinants of health. Lancet (London, England), 401, 1194–1213. 36966782 10.1016/S0140-6736(23)00013-2

[CIT0054] Gislason, M. K., Kennedy, A. M. and Witham, S. M. (2021) The interplay between social and ecological determinants of mental health for children and youth in the climate crisis. International Journal of Environmental Research and Public Health, 18, 4573. 33925907 10.3390/ijerph18094573 PMC8123462

[CIT0055] Goldsmith, L., Raditz, V. and Méndez, M. (2022) Queer and present danger: understanding the disparate impacts of disasters on LGBTQ+ communities. Disasters, 46, 946–973. 34498778 10.1111/disa.12509

[CIT0056] Government of Canada. (2020) Air Pollution: Drivers and Impacts.https://www.canada.ca/en/environment-climate-change/services/environmental-indicators/air-pollution-drivers-impacts.html (last accessed 29 March 2024).

[CIT0057] Government of Ontario. (2020) Protecting People and Property: Ontario’s Flooding Strategy. https://files.ontario.ca/mnrf-2020-flood-strategy-en-2020-03-10.pdf (last accessed 29 March 2024).

[CIT0058] Government of Canada. (2023) *Environment and Climate Change Canada Presents the Winter Seasonal Outlook*. December. https://www.canada.ca/en/environment-climate-change/news/2023/12/environment-and-climate-change-canada-presents-the-winter-seasonal-outlook.html

[CIT0059] Green, H., Bailey, J., Schwarz, L., Vanos, J., Ebi, K. and Benmarhnia, T. (2019) Impact of heat on mortality and morbidity in low and middle income countries: a review of the epidemiological evidence and considerations for future research. Environmental Research, 171, 80–91. 30660921 10.1016/j.envres.2019.01.010

[CIT0060] Grignon-Masse, L., Riviere, P. and Adnot, J. (2011) Strategies for reducing the environmental impacts of room air conditioners in Europe. Energy Policy, 39, 2152–2164.

[CIT0061] Hackett, F., Got, T., Kitching, G. T., MacQueen, K. and Cohen, A. (2020) Training Canadian doctors for the health challenges of climate change. The Lancet Planetary Health, 4, e2–e3. 31924524 10.1016/S2542-5196(19)30242-6

[CIT0062] Haman, F., Souza, S. C. S., Castellani, J. W., Dupuis, M. -P., Friedl, K. E., Sullivan-Kwantes, W. et al. (2022) Human vulnerability and variability in the cold: establishing individual risks for cold weather injuries. Temperature (Austin, Tex.), 9, 158–195. 36106152 10.1080/23328940.2022.2044740 PMC9467591

[CIT0063] Harymann, M., Ogden, N., Lindsay, R., Lawless, V., Deilgat, M. and Sternthal, S. (2014) Lyme disease: summary of the public health agency of Canada’s action plan on Lyme disease. Canada Communicable Disease Report, 40, 88–90. 29769887 10.14745/ccdr.v40i05a03 PMC5864454

[CIT0064] Health Canada. (2020) Reducing Urban Heat Islands to Protect Health in Canada. https://www.canada.ca/en/services/health/publications/healthy-living/reducing-urban-heat-islands-protect-health-canada.html (last accessed 29 March 2024).

[CIT0065] Hill, H., Hadarits, M., Rieger, R., Strickert, G., Davies, E. G. and Strobbe, K. M. (2014) The invitational drought tournament: what is it and why is it a useful tool for drought preparedness and adaptation? Weather and Climate Extremes, 3, 107–116.

[CIT0066] HomeInstead. (2023) 10 Tips to Keep Seniors Safe from Wildfires. June.

[CIT0067] Hopp, S., Dominici, F. and Bobb, J. F. (2018) Medical diagnoses of heat wave-related hospital admissions in older adults. Preventive Medicine, 110, 81–85. 29428173 10.1016/j.ypmed.2018.02.001 PMC6040588

[CIT0068] Hornbostel, V. L., Krell, R. K., Reid, J. J., Schappach, B. L., Volpe, S. and Connally, N. P. (2021) Spray safe, play safe: story-based films increase homeowner confidence about backyard tick management. Journal of Medical Entomology, 58, 857–865. 33225365 10.1093/jme/tjaa230

[CIT0069] Horton, G., Hanna, L. and Kelly, B. (2010) Drought, drying and climate change: emerging health issues for ageing Australians in rural areas. Australasian Journal on Ageing, 29, 2–7. 20398079 10.1111/j.1741-6612.2010.00424.x

[CIT0070] Hyllestad, S., Iversen, A., MacDonald, E., Amato, E., Borge, B. A. S., Bøe, A. et al. (2020) Large waterborne Campylobacter outbreak: use of multiple approaches to investigate contamination of the drinking water supply system, Norway, June 2019. Euro Surveillance, 25, 1–10, 10.2807/1560-7917.ES.2020.25.35.2000011 PMC7472686 32885779

[CIT0071] Ishigami, A., Hajat, S., Kovats, R. S., Bisanti, L., Rognoni, M., Russo, A. et al. (2008) An ecological time-series study of heat-related mortality in three European cities. Environmental Health, 7, 5. 18226218 10.1186/1476-069X-7-5 PMC2266730

[CIT0072] Islam, N. and Winkel, J. (2017) Climate Change and Social Inequality. United Nations, New York.

[CIT0073] Johnson, K. O., Nelder, M. P., Russell, C., Li, Y., Badiani, T., Sander, B. et al. (2018) Clinical manifestations of reported Lyme disease cases in Ontario, Canada: 2005–2014. PLoS One, 13, e0198509. 29856831 10.1371/journal.pone.0198509 PMC5983483

[CIT0074] Karataş, A. and Karataş, E. (2016) Environmental education as a solution tool for the prevention of water pollution. Journal of Survey in Fisheries Sciences, 3, 61–70.

[CIT0075] Kendall, P. A., Val Hillers, V., Medeiros, L. C. (2006) Food safety guidance for older adults. Clinical Infectious Diseases, 42, 91298–91304. 10.1086/503262 16586390

[CIT0076] Kim, J. -S., Kug, J. -S., Jeong, S. -J., Huntzinger, D. N., Michalak, A. M., Schwalm, C. R. et al. (2017) Reduced North American terrestrial primary productivity linked to anomalous Arctic warming. Nature Geoscience, 10, 572–576.

[CIT0077] Kim, Y. -O., Lee, W., Kim, H. and Cho, Y. (2020) Social isolation and vulnerability to heatwave-related mortality in the urban elderly population: a time-series multi-community study in Korea. Environment International, 142, 105868. 32593050 10.1016/j.envint.2020.105868

[CIT0078] Klinenberg, E., Araos, M. and Koslov, L. (2020) Sociology and the climate crisis. Annual Review of Sociology, 46, 649–669.

[CIT0079] Lake, I. R. (2017) Food-borne disease and climate change in the United Kingdom. Environmental Health, 16, 53–59. 29219100 10.1186/s12940-017-0327-0 PMC5773878

[CIT0080] Lepeule, J., Bind, M. -A. C., Baccarelli, A. A., Koutrakis, P., Tarantini, L., Litonjua, A. et al. (2014) Epigenetic influences on associations between air pollutants and lung function in elderly men: the normative aging study. Environmental Health Perspectives, 122, 566–572. 24602767 10.1289/ehp.1206458 PMC4050500

[CIT0081] Lewis, J. and Dickson, J. (2019) *Report on Climate Change Shows Canada Warming at Twice the Rat**e of Rest of World*. https://www.cnn.com/2019/04/01/health/canada-global-warming/index.html#:~:text=Canada%20is%20warming%20up%20faster,the%20rate%20of%20global%20warming (last accessed 29 March 2024).

[CIT0082] Li, M., Gu, S., Bi, P., Yang, J. and Liu, Q. (2015) Heat waves and morbidity: current knowledge and further direction – a comprehensive literature review. International Journal of Environmental Research and Public Health, 12, 5256–5283. 25993103 10.3390/ijerph120505256 PMC4454966

[CIT0083] Lieber, M., Chin-Hong, P., Kelly, K., Dandu, M. and Weiser, S. D. (2022) A systematic review and meta-analysis assessing the impact of droughts, flooding, and climate variability on malnutrition. Global Public Health, 17, 68–82. 33332222 10.1080/17441692.2020.1860247 PMC8209118

[CIT0084] Lubik, A. and Kosatsky, T. (2017) *Developing a Municipal Heat Response Plan: A Guide for Mediumsized Municipalities*. http://www.bccdc.ca/resource-gallery/Documents/Guidelines%20and%20Forms/Guidelines%20and%20Manuals/Health-Environment/Developing%20a%20municipal%20heat%20response%20plan.pdf (last accessed 29 March 2024).

[CIT0085] Martin, S. L., Cakmak, S., Hebbern, C. A., Avramescu, M. -L. and Tremblay, N. (2012) Climate change and future temperature-related mortality in 15 Canadian cities. International Journal of Biometeorology, 56, 605–619. 21597936 10.1007/s00484-011-0449-y

[CIT0086] Meade, R. D., Akerman, A. P., Notley, S. R., McGinn, R., Poirier, P., Gosselin, P. et al. (2020) Physiological factors characterizing heat-vulnerable older adults: a narrative review. Environment International, 144, 105909. 32919284 10.1016/j.envint.2020.105909

[CIT0087] Milman, O. (2023) Climate crisis made spate of Canada wildfires twice as likely, scientists find. The Guardian, August.

[CIT0088] Moffat, H. and Struck, S. (2011) Water-borne Disease Outbreaks in Canadian Small Drinking Water Systems. Small Drinking Water Systems Project. National Collaborating Centres for Public Health, November.

[CIT0089] Moon, K. A., Pollak, J., Poulsen, M. N., Hirsch, A. G., DeWalle, J., Heaney, C. D. et al. (2019) Peridomestic and community-wide landscape risk factors for Lyme disease across a range of community contexts in Pennsylvania. Environmental Research, 178, 108649. 31465993 10.1016/j.envres.2019.108649

[CIT0090] Muscedere, J. and Heckman, G. (2019) *Island Voices: Climate Change Puts the Elderly at Healt**h Risk*. https://www.timescolonist.com/islander/island-voices-climate-change-puts-the-elderly-at-health-risk-4674599 (last accessed 29 March 2024).

[CIT0091] Newell, R. G. (2021) Federal climate policy 101: reducing emissions. Resources for the Future.

[CIT0092] Nhamo, L., Mabhaudhi, T. and Modi, A. (2019) Preparedness or repeated short-term relief aid? Building drought resilience through early warning in southern Africa. Water SA, 45, 1.

[CIT0093] Nova Scotia. (2023), Province Announces Restrictions During Wildfires. Nova Scotia Government, Nova Scotia. https://news.novascotia.ca/en/2023/05/30/province-announces-restrictions-during-wildfires#:~:text=As%20fire%20crews%20battle%20wildfires,priority%2C”%20said%20Minister%20Rushton (last accessed 29 March 2024).

[CIT0094] O’Neill, M. S., Carter, R., Kish, J. K., Gronlund, C. J., White-Newsome, J. L., Manarolla, X. et al. (2009) Preventing heat-related morbidity and mortality: new approaches in a changing climate. Maturitas, 64, 98–103. 19748195 10.1016/j.maturitas.2009.08.005 PMC2793324

[CIT0095] Organization, W.H. (2019), Regional Plan of Action 2019-2023 for Implementation of the Global Vector Control Response 2017-2030. World Health Organization. Regional Office for the Eastern Mediterranean.

[CIT0096] Oudin Åström, D., Åström, C., Forsberg, B., Vicedo-Cabrera, A. M., Gasparrini, A., Oudin, A. et al. (2020) Heat wave-related mortality in Sweden: a case-crossover study investigating effect modification by neighbourhood deprivation. Scandinavian Journal of Public Health, 48, 428–435. 30253698 10.1177/1403494818801615 PMC6713612

[CIT0097] Patel, K., Greenwald, B. D. and Sabini, R. C. (2021) Rehabilitation outcomes in subjects with West Nile neuro-invasive disease. Brain Sciences, 11, 101253. 10.3390/brainsci11101253 PMC8533846 34679318

[CIT0098] Patrick, R., Capetola, T., Townsend, M. and Nuttman, S. (2012) Health promotion and climate change: exploring the core competencies required for action. Health Promotion International, 27, 475–485. 21914637 10.1093/heapro/dar055

[CIT0099] Pelletier, J. (2017) Gouvernance territoriale des risques naturels au Québec et événements extrêmes: le cas de l’inondation à Saint-Jean-sur-Richelieu en 2011. Université du Québecà Montréal, Montreal, Canada.

[CIT0100] Perez, F. P., Perez, C. A. and Chumbiauca, M. N. (2022) Insights into the social determinants of health in older adults. Journal of Biomedical Science and Engineering, 15, 11261. 10.4236/jbise.2022.1511023 PMC9681180 36419938

[CIT0101] Perrotta, K. (2019) Climate change toolkit for health professionals. Canadian Association of Physicians for the Environment (CAPE). https://cape.ca/blog-health-professionals/

[CIT0102] Prek, M. (2004) Environmental impact and life cycle assessment of heating and air conditioning systems, a simplified case study. Energy and Buildings, 36, 1021–1027.

[CIT0103] Prior, T. and Eriksen, C. (2013) Wildfire preparedness, community cohesion and social–ecological systems. Global Environmental Change, 23, 1575–1586.

[CIT0104] Public Health Agency of Canada. (2018) West Nile Virus and Other Mosquito-Borne Diseases Surveillance Report: Annual Edition (2018), Ottawa, Canada. https://www.canada.ca/en/public-health/services/publications/diseases-conditions/west-nile-virus-other-mosquito-borne-diseases-surveillance-annual-report-2018.html (last accessed 29 March 2024).

[CIT0105] Public Health Agency of Canada. (2019) Emergency Management Strategy for Canada: Toward a Resilient 2030. Ottawa, Canada. https://www.publicsafety.gc.ca/cnt/rsrcs/pblctns/mrgncy-mngmnt-strtgy/mrgncy-mngmnt-strtgy-en.pdf (last accessed 29 March 2024).

[CIT0106] Radisic, S. and Newbold, B. (2015) Air quality and health education to increase knowledge and encourage health protective behaviour among older adults in Hamilton, Canada. Environmental Health Review, 58, 87–94.

[CIT0107] Ramadan, A. M. H. and Ataallah, A. G. (2021) Are climate change and mental health correlated? General Psychiatry, 34, e100648. 34825128 10.1136/gpsych-2021-100648 PMC8578975

[CIT0108] Rantakokko, M., Mänty, M. and Rantanen, T. (2013) Mobility decline in old age. Exercise and Sport Sciences Reviews, 41, 19–25. 23038241 10.1097/JES.0b013e3182556f1e

[CIT0109] Reid, C. E., O’neill, M. S., Gronlund, C. J., Brines, S. J., Brown, D. G., Diez-Roux, A. V. et al. (2009) Mapping community determinants of heat vulnerability. Environmental Health Perspectives, 117, 1730–1736. 20049125 10.1289/ehp.0900683 PMC2801183

[CIT0110] Rosenthal, A., Stover, E. and Haar, R. J. (2021) Health and social impacts of California wildfires and the deficiencies in current recovery resources: an exploratory qualitative study of systems-level issues. PLoS One, 16, e0248617. 33770088 10.1371/journal.pone.0248617 PMC7997008

[CIT0111] Royé, D., Codesido, R., Tobías, A. and Taracido, M. (2020) Heat wave intensity and daily mortality in four of the largest cities of Spain. Environmental Research, 182, 109027. 31884190 10.1016/j.envres.2019.109027

[CIT0112] Sahni, V., Scott, A. N., Beliveau, M., Varughese, M., Dover, D. C. and Talbot, J. (2016) Public health surveillance response following the southern Alberta floods, 2013. Canadian Journal of Public Health, 107, e142–e148. 27526210 10.17269/cjph.107.5188 PMC6972453

[CIT0113] Salvador, C., Nieto, R., Linares, C., Díaz, J., Alves, C. A. and Gimeno, L. (2021) Drought effects on specific-cause mortality in Lisbon from 1983 to 2016: risks assessment by gender and age groups. The Science of the Total Environment, 751, 142332. 33182008 10.1016/j.scitotenv.2020.142332

[CIT0114] Salvador, C., Vicedo-Cabrera, A. M., Libonati, R., Russo, A., Garcia, B. N., Belem, L. B. C. et al. (2022) Effects of drought on mortality in macro urban areas of Brazil between 2000 and 2019. GeoHealth, 6, 3e2021GH000534. 10.1029/2021GH000534 PMC8902811 35280229

[CIT0115] Sands, L. P., Do, Q., Du, P., Xu, Y. and Pruchno, R. (2022) Long term impact of Hurricane Sandy on hospital admissions of older adults. Social Science & Medicine (1982), 293, 114659. 34954672 10.1016/j.socscimed.2021.114659 PMC8810733

[CIT0116] Scarciglia, A., Roncucci, L. and Benatti, P. (2023) A West Nile Virus infection expressed as unilateral limb paralysis and complicated by Parsonage–Turner syndrome: a case report. Journal of Medical Case Reports, 17, 54. 36788625 10.1186/s13256-023-03756-w PMC9930236

[CIT0117] Schmeltz, M. T., González, S. K., Fuentes, L., Kwan, A., Ortega-Williams, A. and Cowan, L. P. (2013) Lessons from hurricane sandy: a community response in Brooklyn, New York. Journal of Urban Health: Bulletin of the New York Academy of Medicine, 90, 799–809. 24022182 10.1007/s11524-013-9832-9 PMC3795193

[CIT0118] Scott, J. D. and Scott, C. M. (2018) Lyme disease propelled by *Borrelia burgdorferi*-infected blacklegged ticks, wild birds and public awareness—not climate change. The Journal of Veterinary Medical Science, 6, 1–8.

[CIT0119] Seeman, N., Baker, G. R. and Brown, A. D. (2008) Emergency planning in Ontario’s acute care hospitals: a survey of board chairs. Healthcare Policy, 3, 64. 19305769 PMC2645143

[CIT0120] Sejvar, J. J. (2014) Clinical manifestations and outcomes of West Nile virus infection. Viruses, 6, 606–623. 24509812 10.3390/v6020606 PMC3939474

[CIT0121] Semenza, J. C., McCullough, J. E., Flanders, W. D., McGeehin, M. A. and Lumpkin, J. R. (1999) Excess hospital admissions during the July 1995 heat wave in Chicago. American Journal of Preventive Medicine, 16, 269–277. 10493281 10.1016/s0749-3797(99)00025-2

[CIT0122] Shrubsole, D. (2013) A history of flood management strategies in Canada revisited. Climate Change and Flood Risk Management 95–120.

[CIT0123] Silveira, I. H., Cortes, T. R., Bell, M. L. and Junger, W. L. (2023) Effects of heat waves on cardiovascular and respiratory mortality in Rio de Janeiro, Brazil. PLoS One, 18, e0283899. 37000782 10.1371/journal.pone.0283899 PMC10065291

[CIT0124] Simoni, M., Baldacci, S., Maio, S., Cerrai, S., Sarno, G. and Viegi, G. (2015) Adverse effects of outdoor pollution in the elderly. Journal of Thoracic Disease, 7, 34–45. 25694816 10.3978/j.issn.2072-1439.2014.12.10 PMC4311079

[CIT0125] Singh, S., Hanna, E. G. and Kjellstrom, T. (2015) Working in Australia’s heat: health promotion concerns for health and productivity. Health Promotion International, 30, 239–250. 23690144 10.1093/heapro/dat027

[CIT0126] Smith, B. A. and Fazil, A. (2019) Climate change and infectious diseases: the challenges: how will climate change impact microbial foodborne disease in Canada? Canada Communicable Disease Report, 45, 108–113. 31285700 10.14745/ccdr.v45i04a05 PMC6587690

[CIT0127] Smith, B. A., Meadows, S., Meyers, R., Parmley, E. J. and Fazil, A. (2019) Seasonality and zoonotic foodborne pathogens in Canada: relationships between climate and Campylobacter, *E. coli* and *Salmonella* in meat products. Epidemiology & Infection, 147, e190. 31364535 10.1017/S0950268819000797 PMC6518574

[CIT0128] Stanke, C., Kerac, M., Prudhomme, C., Medlock, J. and Murray, V. (2013) Health effects of drought: a systematic review of the evidence. PLoS Currents, 5, 10.1371/ecurrents.dis.7a2cee9e980f91ad7697b570bcc4b004 PMC3682759 23787891

[CIT0129] Statistics Canada. (2011) *Seniors*. https://www150.statcan.gc.ca/n1/pub/11-402-x/2011000/chap/seniors-aines/seniors-aines-eng.htm

[CIT0130] Statistics Canada. (2012) *Living Arrangements of Seniors*. https://www12.statcan.gc.ca/census-recensement/2011/as-sa/98-312-x/98-312-x2011003_4-eng.cfm

[CIT0131] Statistics Canada. (2017) *Working Seniors in Canada*. https://www12.statcan.gc.ca/census-recensement/2016/as-sa/98-200-x/2016027/98-200-x2016027-eng.cfm

[CIT0132] Statistics Canada. (2021) Air Conditioners. https://www150.statcan.gc.ca/t1/tbl1/en/tv.action?pid=3810001901 (last accessed 29 March 2024).

[CIT0133] Sutherst, R. W. (2004) Global change and human vulnerability to vector-borne diseases. Clinical Microbiology Reviews, 17, 136–173. 14726459 10.1128/CMR.17.1.136-173.2004 PMC321469

[CIT0134] Taylor, J., Symonds, P., Heaviside, C., Chalabi, Z., Davies, M. and Wilkinson, P. (2021) Projecting the impacts of housing on temperature-related mortality in London during typical future years. Energy and Buildings, 249, 111233. 10.1016/j.enbuild.2021.111233 PMC8593871 34819713

[CIT0135] Taylor, J., Symonds, P., Wilkinson, P., Heaviside, C., Macintyre, H., Davies, M. et al. (2018) Estimating the influence of housing energy efficiency and overheating adaptations on heat-related mortality in the West Midlands, UK. Atmosphere, 9, 190.

[CIT0136] Thomas, M. K., Murray, R., Flockhart, L., Pintar, K., Pollari, F., Fazil, A. et al. (2013) Estimates of the burden of foodborne illness in Canada for 30 specified pathogens and unspecified agents, circa 2006. Foodborne Pathogens and Disease, 10, 639–648. 23659355 10.1089/fpd.2012.1389 PMC3696931

[CIT0137] Thomas, S. and Daube, M. (2023) New times, new challenges for health promotion. Health PromotionInternational, 38, 1–3. 10.1093/heapro/daad012 36811825

[CIT0138] Thomas, S., Francis, J., Hennessy, M., Frazer, K., Godziewski, C., Douglass, C. et al. (2024) The year in review—Health Promotion International 2023. Health Promotion International, 39, 1–9. 10.1093/heapro/daad181 38211952

[CIT0139] Thompson, M. P., Norris, F. H. and Hanacek, B. (1993) Age differences in the psychological consequences of Hurricane Hugo. Psychology and Aging, 8, 4606. 10.1037//0882-7974.8.4.606 8292289

[CIT0140] Thomson, M.C. and Stanberry, L.R. (2022) Climate change and vectorborne diseases. NewEngland Journal of Medicine, 387, 1969–1978, 10.1056/NEJMra2200092 36416768

[CIT0141] Tian, L., Qiu, H., Sun, S., Tsang, H., Chan, K. -P. and Leung, W. K. (2017) Association between emergency admission for peptic ulcer bleeding and air pollution: a case-crossover analysis in Hong Kong’s elderly population. The Lancet Planetary Health, 1, e74–e81. 29851584 10.1016/S2542-5196(17)30021-9

[CIT0142] To, P., Eboreime, E. and Agyapong, V. I. (2021) The impact of wildfires on mental health: a scoping review. Behavioral Sciences. 11, 126. 34562964 10.3390/bs11090126 PMC8466569

[CIT0143] Tymstra, C., Stocks, B. J., Cai, X. and Flannigan, M. D. (2020) Wildfire management in Canada: review, challenges and opportunities. Progress in Disaster Science, 5, 100045.

[CIT0144] United Nations. (2017) *Climate Change Impacts Human Health*. https://www.google.com/search?client=safari&rls=en&q=Climate+Change+Impacts+Human+health&ie=UTF-8&oe=UTF-8

[CIT0145] van Steen, Y., Ntarladima, A. -M., Grobbee, R., Karssenberg, D. and Vaartjes, I. (2019) Sex differences in mortality after heat waves: are elderly women at higher risk? International Archives of Occupational and Environmental Health, 92, 37–48. 30293089 10.1007/s00420-018-1360-1

[CIT0146] Vins, H., Bell, J., Saha, S. and Hess, J. J. (2015) The mental health outcomes of drought: a systematic review and causal process diagram. International Journal of Environmental Research and Public Health, 12, 13251–13275. 26506367 10.3390/ijerph121013251 PMC4627029

[CIT0147] Watts, N., Adger, W. N., Agnolucci, P., Blackstock, J., Byass, P., Cai, W. et al. (2015) Health and climate change: policy responses to protect public health. The Lancet, 386, 100061861–100061914. 10.1016/S0140-6736(15)60854-6 26111439

[CIT0148] Westwood, R. (2017) *Canada’s Not Ready for a Future of Massive Storms*. Maclean’s, September.

[CIT0149] WHO. (2014) Climate Change and Health. https://www.who.int/news-room/fact-sheets/detail/climate-change-and-health (last accessed 29 March 2024).

[CIT0150] Wilk, P., Gunz, A., Maltby, A., Ravichakaravarthy, T., Clemens, K. K., Lavigne, E. et al. (2021) Extreme heat and paediatric emergency department visits in Southwestern Ontario. Paediatrics & Child Health, 26, 305–309. 34336059 10.1093/pch/pxaa096 PMC8318534

[CIT0151] Wu, J., Xiao, J., Li, T., Li, X., Sun, H., Chow, E. P. et al. (2015) A cross-sectional survey on the health status and the health-related quality of life of the elderly after flood disaster in Bazhong city, Sichuan, China. BMC Public Health, 15, 163. 25884807 10.1186/s12889-015-1402-5 PMC4359459

[CIT0152] Yang, J., Yin, P., Sun, J., Wang, B., Zhou, M., Li, M. et al. (2019) Heatwave and mortality in 31 major Chinese cities: definition, vulnerability and implications. The Science of the Total Environment, 649, 695–702. 30176480 10.1016/j.scitotenv.2018.08.332

[CIT0153] Ye, T., Guo, Y., Chen, G., Yue, X., Xu, R., Coêlho, M. S. Z. S. et al. (2021) Risk and burden of hospital admissions associated with wildfire-related PM2 5 in Brazil, 2000–15: a nationwide time-series study. The Lancet Planetary Health, 5, e599–e607. 34508681 10.1016/S2542-5196(21)00173-X

[CIT0154] Yeganeh, A. J., McCoy, A. P. and Schenk, T. (2020) Determinants of climate change policy adoption: a meta-analysis. Urban Climate, 31, 100547.

[CIT0155] Yodsuban, P. and Nuntaboot, K. (2021) Community-based flood disaster management for older adults in southern of Thailand: a qualitative study. International Journal of Nursing Sciences, 8, 409–417. 34631991 10.1016/j.ijnss.2021.08.008 PMC8488803

[CIT0156] Yusa, A., Berry, P., Cheng, J. J., Ogden, N., Bonsal, B., Stewart, R. et al. (2015) Climate change, drought and human health in Canada. International Journal of Environmental Research and Public Health, 12, 8359–8412. 26193300 10.3390/ijerph120708359 PMC4515727

[CIT0157] Zeldin-O’Neill, S. (2019) It’s a crisis, not a change: the six Guardian language changes on climate matters. The Guardian. https://www.theguardian.com/environment/2019/oct/16/guardian-language-changes-climate-environment#:~:text=4%20years%20old-,'It's%20a%20crisis%2C%20not%20a%20change'%3A%20the%20six%20Guardian,language%20changes%20on%20climate%20matters&text=Climate%20change%20is%20no%20longer,broader%20impact%20of%20climate%20change (last accessed 29 March 2024).

[CIT0158] Zhang, H., Liu, L., Zeng, Y., Liu, M., Bi, J. and Ji, J. S. (2021) Effect of heatwaves and greenness on mortality among Chinese older adults. Environmental Pollution (Barking, Essex : 1987), 290, 118009. 34523521 10.1016/j.envpol.2021.118009

[CIT0159] Zhang, K., Chen, T. -H. and Begley, C. E. (2015) Impact of the 2011 heat wave on mortality and emergency department visits in Houston, Texas. Environmental Health, 14, 11. 25627975 10.1186/1476-069X-14-11 PMC4417210

[CIT0160] Zomer, T. P., van Bemmel, T., van Munster, B., van Kooten, B. and Vermeeren, Y. M. (2018) Lyme borreliosis and depressive symptoms in patients aged 65 years and older referred to a tertiary Lyme centre. European Journal of Internal Medicine, 51, e19–e20. 29475771 10.1016/j.ejim.2018.01.030

